# False-seronegative HCV infection motivated by interference with cryoglobulins

**DOI:** 10.1515/almed-2020-0086

**Published:** 2021-04-26

**Authors:** Gemma Recio Comí, Carmen Molina Clavero, Sandra Calabuig Ballester, Clara Benavent Bofill, Ester Picó-Plana, Carla Martín Grau, Cristina Gutiérrez Fornés, Ma Teresa Sans Mateu

**Affiliations:** Clinical Chemistry Laboratory, Catalan Institute of Health (ICS)-Camp de Tarragona-Terres de l’Ebre, Joan XXIII University Hospital in Tarragona, Tarragona, Spain; Clinical Chemistry Laboratory, Catalan Institute of Health (ICS)-Camp de Tarragona-Terres de l’Ebre – Verge de la Cinta Hospital in Tortosa, Tarragona, Spain

**Keywords:** cryoglobulinemia, hepatitis C virus, interference

## Abstract

**Objectives:**

Cryoglobulins (CGs) are serum proteins that undergo a reverse cold-induced precipitation *in vitro*. The CGs are a well-known cause of analytical interferences in several laboratory tests, leading to spurious results. With this in view, we present a case of a patient initially misdiagnosed due to CGs interference in Hepatitis C Virus (HCV) serology.

**Case presentation:**

We report a case of a woman of advanced age affected by acute renal failure that required urgent haemodialysis. In the absence of infections and other causes of CGs production, a diagnosis of acute renal failure secondary to essential cryoglobulinemia was established. However, an unexpected positive HCV viral load was encountered. At this point, a false-seronegative HCV infection conditioned to CGs interference *in vitro* was suspected, confirmed by repeating serology in pre-warmed serum. Finally, the patient was correctly diagnosed with HCV-secondary cryoglobulinemia.

**Conclusions:**

As shown in the case, the presence of CGs in blood may represent a challenge for the correct interpretation of several laboratory tests. The identification of CGs and the pre-treatment of serum are decisive to avoid spurious results and reach a genuine diagnosis.

## Introduction

Laboratory tests play an important role in patients’ diagnosis. Nevertheless, in some clinical situations, laboratory tests can produce false results due to *in vitro* artefacts. Paraproteins are plasma proteins appearing in large quantities because of some pathological condition, that can cause several effects in laboratory parameters [1]. *In vitro* effects of cryoglobulins (CGs) are well known when affecting blood cell counts by haematology analysers. Erroneous white blood cells (WBCs) counts have been reported as the major artefact, as well as abnormal platelet counts and erythrocyte sedimentation rate [2].

The CGs are a group of serum proteins with the physical property of reverse cold-induced precipitation *in vitro*. The CGs are produced by a mature B-cell clonal expansion in disorders such as multiple myeloma, lymphoproliferative disorders, chronic infections or autoimmune diseases. They also can occur in the absence of any apparent relevant disease (essential cryoglobulinemia). Cryoglobulinemia is classified based on CGs composition with the Brouet et al. subgroups, in three types: type I (monoclonal immunoglobulins), type II (monoclonal and polyclonal immunoglobulins) and type III (polyclonal immunoglobulins). Both types II and III are called mixed cryoglobulinemia and are associated with Hepatitis C virus (HCV) infection [3]. The pathogenesis of cryoglobulinemia remains unclear, but the host’s immune response may play an important role. Apparently, the presence of CGs is due to chronic viraemia and to the generation of rheumatoid factor because of the continuous presentation of antigen-immunoglobulin complexes to B-cells [4].

Mixed CGs are detected in approximately 40% of patients with HCV infection, and they are associated with an increased mortality and morbidity. Patients with HCV and CGs are more likely to have cirrhosis and increased fibrosis score than patients without CGs. Besides that, the accumulation of CGs in vessel walls could be responsible for a variety of extrahepatic manifestations, including vasculitis, glomerulonephritis, arthritis, purpura, neuropathies and cerebritis [5].

## Case presentation

We report a case of an 84-year-old woman who was presented to the Nephrology Department affected by acute renal failure and pulmonary oedema that required urgent haemodialysis. Upon routine examination, the patient presented drowsiness and disorientation, but acute pathology or infectious disease was discarded by a cranial computed tomography (CT) and a lumbar puncture. The patient showed Meltzer’s triad, composed of purpuric lesions, arthralgias and weakness. Initial laboratory tests confirmed kidney injury: creatinine 2.44 mg/dL (reference value [RV]: 0.5–1 mg/dL), urea 198 mg/dL (RV: 18–46 mg/dL), potassium 6.1 mmol/L (RV: 3.5–5.1 mmol/L) and sodium 128 mmol/L (RV: 132–146 mmol/L). Haematuria with dysmorphic erythrocytes was visualized in urinary sediment, being proteinuria also present (+2 on a dipstick = 100 mg/dL). Laboratory assays revealed normochromic and normocytic anaemia, and increased leukocyte count with neutrophilia. No abnormal coagulation values were found. Blood and urine cultures were negative. Chest X-ray and abdominal CT were both normal. Renal echography determined no morphological alterations. Kidney biopsy could not be performed considering the patient’s poor general condition. An immunology study was carried out, encountering decreased serum levels of C3 and C4 and increased value for rheumatoid factor. Neither antinuclear antibodies nor antineutrophil cytoplasmic antibodies were found. Kappa/lambda-free light chain ratio and IgM quantification were increased. A discrete band in gamma globulin fraction, composed of IgM kappa, was detected in serum protein electrophoresis and immunofixation. However, marrow bone aspirate discarded blood malignancy. Viral serologies were screened, and all were found to be negative, including HCV (ADVIA Centaur^®^ EIA HCV Assay, Siemens Healthineers). Further laboratory results revealed a type II cryoglobulinemia composed by polyclonal IgG and monoclonal IgM Kappa (cryocrit of 14%). Skin biopsy of petechiae was compatible with medium vessel vasculitis. In the absence of known causes of CGs production, acute renal failure secondary to essential cryoglobulinemia was diagnosed.

As established in dialysis protocol, HCV viral load was analysed (COBAS 4800 HCV 120t, ROCHE Diagnostics^®^), leading to an unexpected result of 3,210,000 IU/mL. The patient had no symptoms, and no liver function biomarkers suggestive of hepatitis. Cross-contamination of samples and window period was discarded. At this point, a false-seronegative HCV infection conditioned to CGs effect *in vitro* was suspected.

HCV serology was repeated on serum kept at 37 °C for 60 min until analysis, to allow the dissolution of putative cryoprecipitates. HCV-antibodies resulted reactive with a 2.68 Index Value (IV) (reactive if IV≥1). Finally, the patient was properly diagnosed with acute renal failure secondary to HCV-induced cryoglobulinemia. Other measures like immunoglobulin levels, C3 and C4 levels, rheumatoid factor, serum protein electrophoresis and immunofixation were repeated with serum heated at 37 °C to avoid spurious results due to CGs interference.

The primary goal in the treatment of a chronic HCV infection is the eradication of the virus with antiviral therapy [6]. Unfortunately, the patient could not be treated because of a sudden incapacity to swallow. The second goal is the treatment of autoimmune features using corticosteroids, Rituximab and/or plasmaphaeresis. The patient was medically unstable, not candidate for aggressive medical care. In view of the severity of the disease and the advanced age, it was decided not to treat the patient with Rituximab or plasma exchange. The treatment was corticosteroid therapy for the extrahepatic manifestations, in addition to haemodialysis and furosemide for the renal failure. The patient showed deterioration with severe rectal bleeding due to multiple vascular lesions and apnoea episodes. Comfort measures were started, and the patient was finally exitus.

The lack of treatment for the chronic HCV infection was not considered the cause of the death. The patient had no symptoms and no abnormal liver function parameters (normal coagulation, normal transaminases, normal size and morphology liver, homogeneous, without focal lesions observed by CT). The patient presented a fatal outcome due to an HCV-secondary multisystemic vasculitis that could not be treated with a more aggressive therapy.

## Discussion

Cryoglobulinemia is a well-known cause of false negative results in the polymerase chain reaction for the detection of HCV-RNA. In this situation, the serology may be positive but viral particles can be masked in the cryoprecipitate [7]. The presented case is the opposite situation. Tini et al. described a similar case in 2007, but they were only able to detect RNA virus in the cryoprecipitate [8].

This case presents a false-negative HCV serology likely caused by CGs interference. As shown here, the presence of CGs in blood may represent a challenge for the correct interpretation of several laboratory tests. Before the discovery of HCV infection, most cases of secondary cryoglobulinemia were defined as essential cryoglobulinemia, without an underlying identifiable cause. Since the association of HCV and CGs was described, the diagnosis of essential cryoglobulinemia has become rare. In this case report, the presence of CGs in blood was the cause of an early misdiagnosis of idiopathic cryoglobulinemia instead of HCV-secondary cryoglobulinemia. This can be explained because of the precipitation of CGs, which traps HCV-antibodies from serum and leads to a false negative result. Pre-warming serum at 37 °C solubilizes the cryoprecipitate and liberates antibodies, allowing their detection, which in this circumstance revealed a previous non-diagnosed HCV infection.

Generally, false negative results are more concerning than false positives. Given the clinical significance of HCV infection, a false seronegative case represents a risk for the patient and for the community itself. On the one hand, a non-diagnosticated patient cannot benefit from antiviral treatment. On the other hand, medical and analytical procedures like haemodialysis can be applied to the patient, causing a risk of nosocomial transmission of the infection.

A highly sensitive assay is crucial to avoid false negative results. The sensitivity of the ADVIA Centaur^®^ EIA HCV Assay reported by Siemens is 100% (95% CI). Many studies were performed by comparing it with alternative CE-marked assays like Architect^®^ Anti-HCV or Elecsys^®^ Anti-HCV. The overall concordance between the Advia Centaur^®^ Anti-HCV and those other assays was satisfactory. Sensitivity of Siemens assay is equivalent to the others and is adequate for the HCV routine screening [9], [10].

Some cases of false negative serologies other than HCV have been described due to serum CGs. Similarly, false positives have been reported (for example, in Chikungunya or syphilis infection) [11], [12]. Some cases of false-seronegative HCV infection results have been reported due to window period or immunosuppressed patients [13]. Enzyme Immunoassays results may be negative in profoundly immunosuppressed patients and in patients on haemodialysis [14]. In this case, window period was discarded and the patient was not receiving immunosuppressive treatment when serologies were done.

## Conclusions

There are relevant analytical interferences are caused by serum paraproteins. This case highlights the importance of investigating the discrepancies in analytical results to reach a genuine diagnosis. Given the clinical significance of HCV infection, a false seronegative case represents an important risk. We hypothesize that cryoglobulinemia secondary to viral infection may be under-diagnosticated due to the trapping effect of the CGs on the viral antibodies. When a serum positive to CGs but negative to viral serologies at room temperature is detected, we propose the application of an algorithm that includes repeating the serologies on pre-warmed serum at 37 °C for 60 min. This incubation step may reduce the interference of CGs. Additionally; screening for HCV-antibodies in combination with HCV-RNA testing appears to be the safest way to prevent that potential active HCV infections scape screening.

## References

[1] King RI, Florkowski CM (2010). How paraproteins can affect laboratory assays: spurious results and biological effects. Pathology.

[2] Fohlen-Walter A, Jacob C, Lecompte T, Lesesve JF (2002). Laboratory identification of cryoglobulinemia from automated blood cell counts, fresh blood samples, and blood films. Am J Clin Pathol.

[3] Damoiseaux J, Cohen Tervaert JW (2014). Diagnostics and treatment of cryoglobulinaemia: it takes two to tango. Clin Rev Allergy Immunol.

[4] Brouet JC, Clauvel JP, Danon F, Klein M, Seligmann M (1974). Biologic and clinical significance of cryoglobulins. A report of 86 cases. Am J Med.

[5] Kayali Z, Labrecque DR, Schmidt WN (2006). Treatment of hepatitis C cryoglobulinemia: mission and challenges. Curr Treat Options Gastroenterol.

[6] Oliver M, Grandadam M, Marimoutou C, Rogier C, Botelho-Nevers E, Tolou H (2009). Persisting mixed cryoglobulinemia in Chikungunya infection. PLoS Neglected Trop Dis.

[7] Van Thiel DH, Caraceni P, Wright HI, Nadir A, Gavaler JS (1995). Cryoglobulinemia: a cause for false negative polymerase chain reaction results in patients with hepatitis C virus positive chronic liver disease. J Hepatol.

[8] Tini GM, Wüscher V, Jeker R (2007). Hepatitis C infection with false negative serology in a patient with mixed cryoglobulinemic vasculitis. Dtsch Med Wochenschr.

[9] Yoo SJ, Wang LL, Ning HC, Tao CM, Hirankarn N, Kuakarn S (2015). Evaluation of the Elecsys® Anti-HCV II assay for routine hepatitis C virus screening of different Asian Pacific populations and detection of early infection. J Clin Virol.

[10] Oh EJ, Chang J, Yang JY, Kim Y, Park YJ, Han K (2013). Different signal-to-cut-off ratios from three automated anti-hepatitis C virus chemiluminescence immunoassays in relation to results of recombinant immunoblot assays and nucleic acid testing. Blood Transfus.

[11] Spearman CW, Dusheiko GM, Hellard M, Sonderup M (2019). Hepatitis C. Lancet.

[12] Jones RR, Pusey C, Schifferli J, Johnston NA (1983). Essential mixed cryoglobulinaemia with false-positive serological tests for syphilis. Br J Vener Dis.

[13] Chamie G, Bonacini M, Bangsberg DR, Stapleton JT, Hall C, Overton ET (2007). Factors associated with seronegative chronic hepatitis C virus infection in HIV infection. Clin Infect Dis.

[14] Pawlotsky JM, Negro F, Aghemo A, Berenguer M, Dalgard O, Dusheiko G (2018). EASL recommendations on treatment of hepatitis C 2018. J Hepatol.

